# Predictive Factors for Patient Recovery Following Triangular Fibrocartilage Foveal Repair Surgery: A Retrospective Case-Series

**DOI:** 10.1177/15589447251325821

**Published:** 2025-03-31

**Authors:** Luke McCarron, Brooke K Coombes, Randy Bindra, Brett Dyer, Steven Watson, Leanne Bisset

**Affiliations:** 1 Griffith University, Southport, QLD, Australia; 2 Bond University, Robina, QLD, Australia; 3 Gold Coast Hospital and Health Service, Southport, QLD, Australia; 4 Australian Research Collaboration on Hands, Mudgeeraba, QLD, Australia

**Keywords:** triangular fibrocartilage, surgery, wrist, ligament repair, rehabilitation

## Abstract

**Background:**

There are many factors that may influence patient recovery following triangular fibrocartilage complex (TFCC) foveal repair surgery. This study aimed to retrospectively analyze patient records following TFCC foveal repair surgery to identify characteristics that predict patient outcomes.

**Methods:**

A multicenter, retrospective case-series was conducted. Informed written consent was obtained from participating hand therapy clinics, who provided deidentified patient records for adult patients following TFCC foveal repair surgery between January 1 2015 and December 31 2020. Predictors of outcomes were identified using Linear Mixed Effects Regression and Logistic Regression models.

**Results:**

A total of 210 patients were included. The most notable improvements in range of motion (ROM) and grip strength, and pain reduction, were observed in the first 10 weeks postsurgery. Longer forearm immobilization duration predicted poorer ROM for pronation, flexion, and extension. Workcover (compensable) patients demonstrated poorer ROM progression compared with private patients. Forty-two patients (20%) required further surgery, of which was due to postoperative TFCC rupture for 22 patients (10%). Patients who received a shorter wrist immobilization period were more likely to experience TFCC rupture. The duration of time between injury and operative treatment did not predict ROM, grip strength, or pain progression.

**Conclusions:**

Longer forearm immobilization predicted poorer ROM and grip strength progression, whereas shorter wrist immobilization predicted an increased risk of TFCC rupture. These findings support a staggered commencement of wrist and forearm ROM exercises, whereby forearm rotation exercises could commence earlier than wrist exercises. The duration of time between injury and operative treatment did not predict ROM, grip strength, or pain progression.

## Introduction

The triangular fibrocartilage complex (TFCC) is a critical structure located on the ulnar aspect of the wrist. The primary role of the TFCC is stabilization of the distal radioulnar joint (DRUJ), which is crucial to achieve the complex movements required of the hand and wrist.^1,2^ The TFCC can be prone to injury, particularly in athletes or due to trauma, which can lead to pain, instability, and decreased wrist and forearm range of motion (ROM).^3,4^ Treatment following TFCC injury may include surgery to reattach the disrupted ligament to its insertion point (fovea), especially when DRUJ instability is present.^5^

Following TFCC foveal repair surgery, postoperative rehabilitation is considered necessary to achieve optimal outcomes. Previous research has explored the various rehabilitation characteristics following TFCC foveal repair surgery, highlighting notable differences between the recommendations provided by hand therapists^6^ and wrist and hand surgeons.^7^ For example, variations in immobilization duration ranged from “Not Required” to “8 weeks,” while ROM exercise commencement time also varied from “Immediately” to “Later than 8 weeks.” To date there have been no direct comparison of TFCC rehabilitation regimens, hence there is no consensus of the optimal period of restricted motion or timing of commencement of loading. There is some evidence that early mobilization may result in better outcomes following ligament reconstruction;^8,9^ however, the benefits of early mobilization following TFCC foveal repair surgery are currently unknown.

Patient and surgical factors, such as age, mechanism of injury (MOI), occupation, Workcover status (Workers Compensation), and surgical procedure, may influence recovery following TFCC foveal repair surgery, as is well documented in other upper limb surgeries.^10^ In addition, patients receiving compensation through Workcover have been observed to take more than twice as long to recover following wrist surgery, such as scaphoid internal fixation.^11^ However, it is currently unclear whether patient demographic, surgical, or rehabilitation characteristic factors can predict recovery following TFCC foveal repair surgery. Furthermore, the trajectory of recovery after TFCC foveal repair surgery is also unknown for factors such as ROM, grip strength, or pain.

### Aims and Objectives

The aim of this study was to describe predictors of outcomes following TFCC foveal repair surgery. Specific objectives were: (1) to document the trajectory of progression of ROM, grip strength, and pain; and (2) to identify whether rehabilitation or surgical characteristics predicted patient outcomes over time.

## Methods

A multicenter, retrospective case-series was conducted, with institutional ethical approval granted (GU Ref No: 2022/557). Private practice hand therapy clinic stakeholders from around the nation were invited to voluntarily participate at the annual national scientific conference 2021 and 2022. Ten clinic stakeholders reported interest, 7 of which had capacity to participate. Informed written consent was obtained from all clinic stakeholders (data custodians) prior to commencement. The data custodians identified the relevant therapy records from their respective patient databases and provided a deidentified copy to LM. The inclusion criteria were adult patients (18 years or older at the time of surgery), who had undergone TFCC foveal repair surgery between January 1, 2015 and December 31, 2020. There was no minimum therapy participation for inclusion. Patients with other procedures, such as TFCC debridement, radioulnar joint reconstruction, or arthroplasty, were excluded.

Two members of the research team (LM and SW) independently extracted predetermined data variables from the patient records using an Excel spreadsheet (Microsoft, IBM, USA), with a third author (LB) to settle any disagreements. Operative records were not reviewed. The data custodians could be contacted by the research team to request clarification if the data were ambiguous.

The following independent variables (candidate prognostic factors) were extracted where available. Demographic variables included sex (male or female), age at the time of surgery (years), occupation (categorized using the Australian Standard Classifications of Occupations Job Classification Guide),^12^ and compensable claim status (Workcover or Private). Injury variables included the side of injury (right or left), dominant or nondominant side affected, MOI, and time between the date of injury and date of surgery. Surgical variables included TFCC repair only (single procedure) or TFCC repair with at least one other secondary procedure (multiprocedure). Rehabilitation variables included immobilization method (orthosis/cast/brace), immobilization duration (defined by the length of time the patient was instructed to wear the immobilization method) extracted separately for the wrist and forearm, and ROM exercise commencement time also extracted separately for wrist (extension/flexion, ulnar/radial deviation), forearm (supination/pronation (SUP/PRO)), and elbow (extension/flexion).

The following dependent variables of interest were extracted where available. Continuous variables included wrist and forearm ROM (degrees), grip strength (kilograms), and pain intensity (numerical rating scale 0-10). The data were grouped relative to the postoperative week, which was determined at the time of extraction using an online date-difference calculator.^13^ Data were averaged if more than one treatment session was provided per week. The presence of postoperative complications, such as documented TFCC rupture or additional surgery, was considered as dichotomous outcome (yes/no). Time to discharge was defined as the time between date of surgery and date of discharge from hand therapy.

### Statistical Analysis

The PROGnostic RESearch Strategy framework was implemented to guide the statistical analysis process.^14^ Patient data were exported from Excel into SPSS Version 29.0.1.0^15^ (IBM Corporation, USA) and RStudio Version 1.2.5033 (Posit PBC, USA) for analysis.^16^ Visual inspection for out-of-range values was completed with none identified. Descriptive statistics were presented in terms of frequency and percentage, or median and interquartile range (IQR). Linear Mixed Effects Models^17^ were used to model the association between recovery time and each continuous outcome (ROM, grip strength, pain), and were also used to assess the relationship between individual candidate prognostic factors and each outcome. Splines were used in the linear mixed models to account for the changes in trajectories during the different stages of recovery. A Linear Mixed Effects Model was chosen to allow for heterogenous measurement time points, allowing for group analysis while simultaneously accounting for individual patient variability.^17^ Due to this, the comparison is not specific to a single time point, but rather reflects the estimated mean difference between groups across all measured time points. A Logistic Regression Model^18^ was used to assess the relationship between the binary outcome TFCC rupture and each of the candidate prognostic variables. For all models, age and sex were adjusted for, to determine whether the candidate prognostic factors were predictive of the outcome over and above known nonmodifiable factors. The most common immobilization method was used as the comparator for analysis. Significance was set to .05 with odds ratios and 95% confidence intervals presented. The Strengthening the Reporting of Observational studies in Epidemiology guidelines were followed to improve the quality, transparency, and reproductivity of this study.^19^

## Results

Data from a total of 210 patients were included in this study. Demographic, injury, and surgical characteristics of this sample are summarized in Table 1. The median patient age was 39 (IQR 29-50) years, with a similar proportion of male (51%) and female patients (49%). The most frequently reported MOI was a fall onto an outstretched hand, accounting for 35.5% of cases, followed by “overuse” injuries at 17%. The predominant occupational categories among patients were professionals/associate professionals and tradespersons (Supplemental Table 1). The mean duration between the date of injury and the date of surgery was 65 weeks, with a range from 1 week to 4.3 years. The most common combination of procedures performed included TFCC repair with ulnar-shortening osteotomy (21 patients) and TFCC repair with carpal tunnel release (10 patients; Supplemental Table 2).

**Table 1.** Patient Demographic, Injury, and Surgical Characteristics of the Sample (n = 210).

Characteristics	Frequency (%) or median^a^ [interquartile range]
Sex	
Male	107 (51)
Female	103 (49)
Age at date of surgery	
Years^a^	39 [29-50]
Occupation	
Managers and administrators	11 (5)
Professionals	37 (17.5)
Associate professions	35 (16.5)
Tradespersons and related workers	34 (16)
Advanced clerical and service workers	15 (7)
Clerical, sales, and service workers	3 (1.5)
Production and transport workers	4 (2)
Elementary clerical, sales, and service workers	22 (10.5)
Laborers and related workers	10 (5)
Nil—retired or not employed	20 (9.5)
Homecare or housewife	2 (1)
Student	17 (8)
Dominant hand	
Right	187 (89)
Left	23 (11)
Dominant affected	
Dominant affected	114 (54)
Nondominant affected	96 (46)
Insurance status	
Workcover (workers compensation)	109 (52)
Private insurance	101 (48)
Mechanism of injury	
FOOSH	75 (35.5)
Overuse	36 (17)
Heavy lifting	33 (15.5)
Forced twisting	20 (9.5)
Motor vehicle accident	12 (6)
Distal radius fracture	10 (4.5)
Fall from height	6 (3)
Motor bike accident	6 (3)
Altercation	4 (2)
Direct impact	4 (2)
Punch injury	2 (1)
Pushing heavy object	2 (1)
Surgical procedure	
Single procedure (TFCC repair)	125 (60)
Multiple procedure (TFCC repair + secondary)	85 (40)
Time from injury to surgery, wk^a^	25 [13-60]
Range	1-1574 wk (4.3 y)

*Note.* A student group was added to represent high school or university/college students who met the inclusion criteria. FOOSH = fall onto an outstretched hand; TFCC = triangular fibrocartilage complex.

a indicates median [interquartile range].

### Rehabilitation Characteristics

All 210 patients received 1 of 8 different immobilization methods postsurgery, 4 that immobilized the wrist and forearm (Sugartong thermoplastic orthosis (TPO), above elbow cast, Munster TPO, forearm-based ulnar gutter TPO) and 4 that predominately immobilized the wrist only (forearm-based volar TPO, wrist brace, forearm-based circumferential wrist TPO, short-arm cast). The most common immobilization method was the Sugartong TPO (77 patients, 36.5%), followed by an above elbow cast (67 patients, 32%).

Immobilization duration and exercise commencement times are illustrated in Supplemental Figures 1 and 2. Wrist immobilization ranged from 2 to 17 weeks, whereas forearm immobilization ranged from 1 to 10 weeks. Six weeks was most common duration for wrist (40%) and forearm (48%) immobilization. Range of motion exercise commencement time ranged from 1 to 10 weeks for wrist extension/wrist flexion (WE/WF), ulnar deviation/radial deviation (UD/RD), forearm SUP/PRO, and elbow extension/elbow flexion. The most common commencement week was 6 weeks for WE/WF (49%), UD/RD (49%), and SUP/PRO (48%), whereas elbow motion was commonly commenced in the first week postsurgery (106 patients, 50%).

### ROM, Grip Strength, and Pain

**Figure 1.**
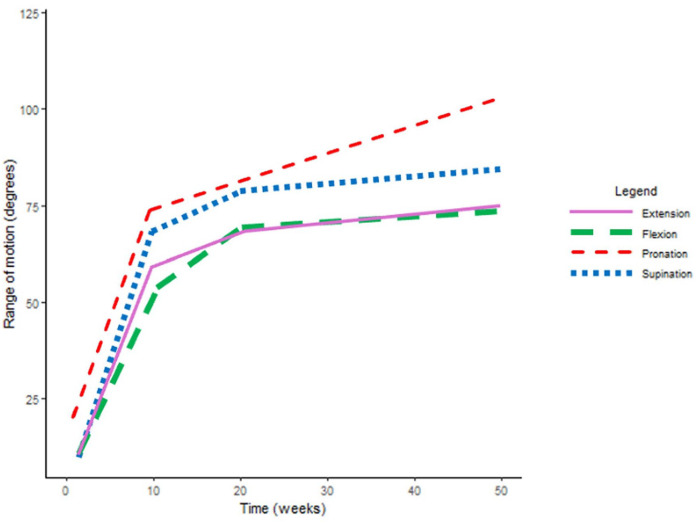
Mean population trajectories for range of motion (degrees per week). This figure demonstrates the mean population trajectories for range of motion throughout recovery, represented as degrees per week, for wrist extension, wrist flexion, forearm supination, and forearm pronation, respectively.

**Figure 2.**
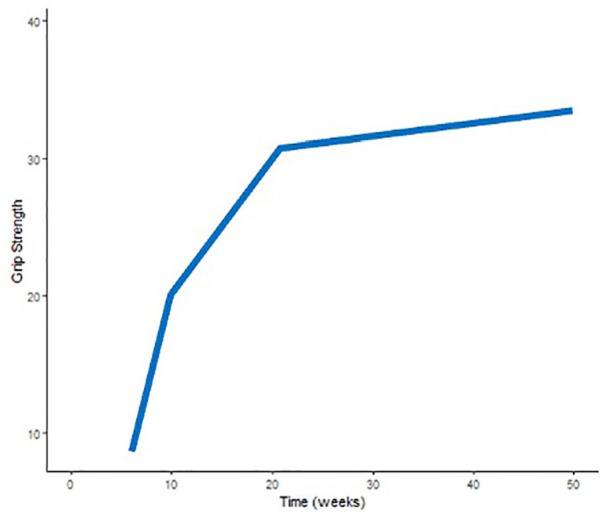
Mean population trajectory for grip strength (mean kilograms per week). This figure demonstrates the mean population trajectory for grip strength throughout recovery, represented as kilograms per week and measured using a standard Jamar dynamometer.

**Figure 3.**
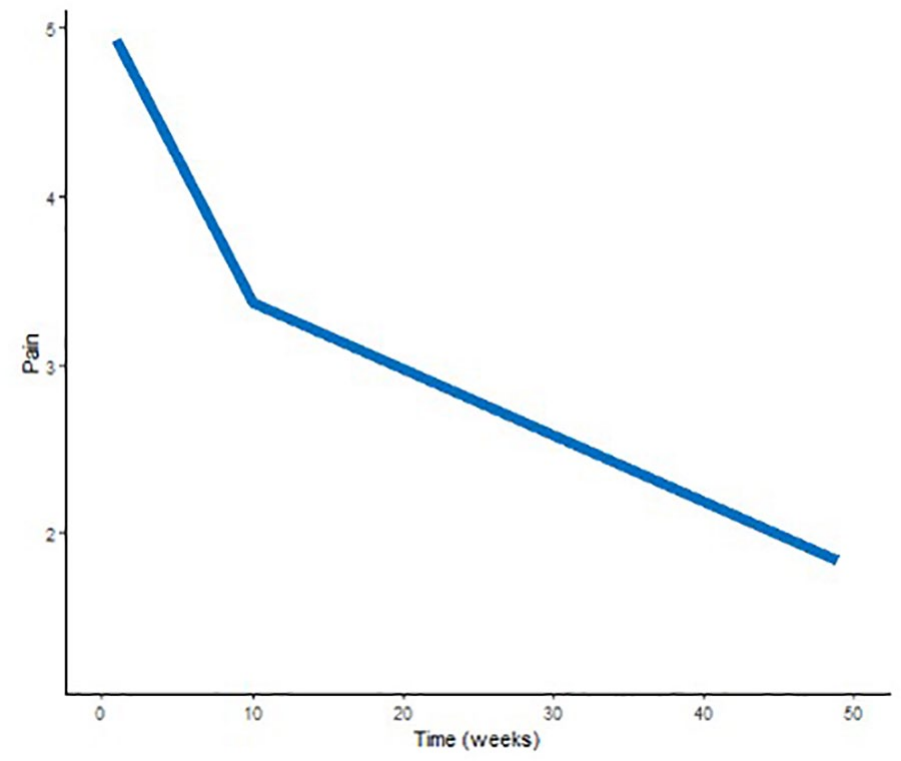
Mean population trajectory for pain (mean pain rating per week). This figure demonstrates the mean population trajectory for pain throughout recovery, represented as the mean numerical rating score.

The mean improvement in ROM, grip strength, and pain per week (with significance and 95% CI) is reported in Table 2, while Figures 1-3 illustrate the mean trajectories for the sample. For the outcomes ROM, grip strength, and pain, the most notable improvements were observed in the first 10 weeks postsurgery.

**Table 2.** Change in Trajectory of Range of Motion, Grip Strength, and Pain During the Different Stages of Recovery According to the Linear Mixed Effects Model.

Movement plane	Mean improvement, per wk	Significance, Pr(>|*t*|)	95% confidence interval
Wrist extension			
0-10 wk	5.4°	<.001	4.9-5.9
11-20 wk	0.8°	<.001	0.6-1.1
>20 wk	0.2°	.02	0.1-0.4
Wrist flexion			
0-10 wk	4.7°	<.001	4.5-5.0
11-20 wk	1.6°	<.001	1.2-1.9
>20 wk	0.1°	.07	0.01-0.2
Supination			
0-10 wk	6.6°	<.001	5.7-7.5
11-20 wk	0.9°	<.001	0.5-1.3
>20 wk	0.2°	.003	0.1-0.3
Pronation			
0-10 wk	5.9°	<.001	5.1-6.8
11-20 wk	0.7°	<.001	0.5-0.9
Grip strength			
0-10 wk	2.9 kg	<.001	2.4-3.4
11-15 wk	1.3 kg	<.001	1.0-1.6
16-20 wk	0.7 kg	<.001	0.4-0.99
>20 wk	0.1 kg	.02	0.02-0.2
Pain			
0-10 wk	−1.7 units	.01	−2.9 to −0.5
>10 wk	−0.4 units	.09	−0.8 to −0.1

### Adverse Events

Additional surgery was reported for 42 patients (20%), with postoperative TFCC rupture documented as the indication for surgery in 22 patients (10%). Other reasons for surgery included pain, weakness, or paresthesia. Additional surgeries include TFCC rerepair (n = 16), neurolysis (n = 5), excision of pisiform (n = 3), removal of metalwork (n = 2), DRUJ arthroplasty (n = 2), removal of suture knot (n = 1), Extensor Carpi Ulnaris stabilization (n = 1), ulnar-shortening osteotomy (n = 1), Extensor Digiti Minimi tenolysis (n = 1), or a combination of the above procedures (n = 10). Triangular fibrocartilage complex rerepair was completed for 16 of the 22 patients with reported TFCC rupture (73%), with 6 patients reported as “unrepairable” and receiving an alternate procedure, such as DRUJ arthroplasty. Complex regional pain syndrome was reported for 3 patients (1.5%).

### Candidate Prognostic Factors for ROM Progression

**Figure 4.**
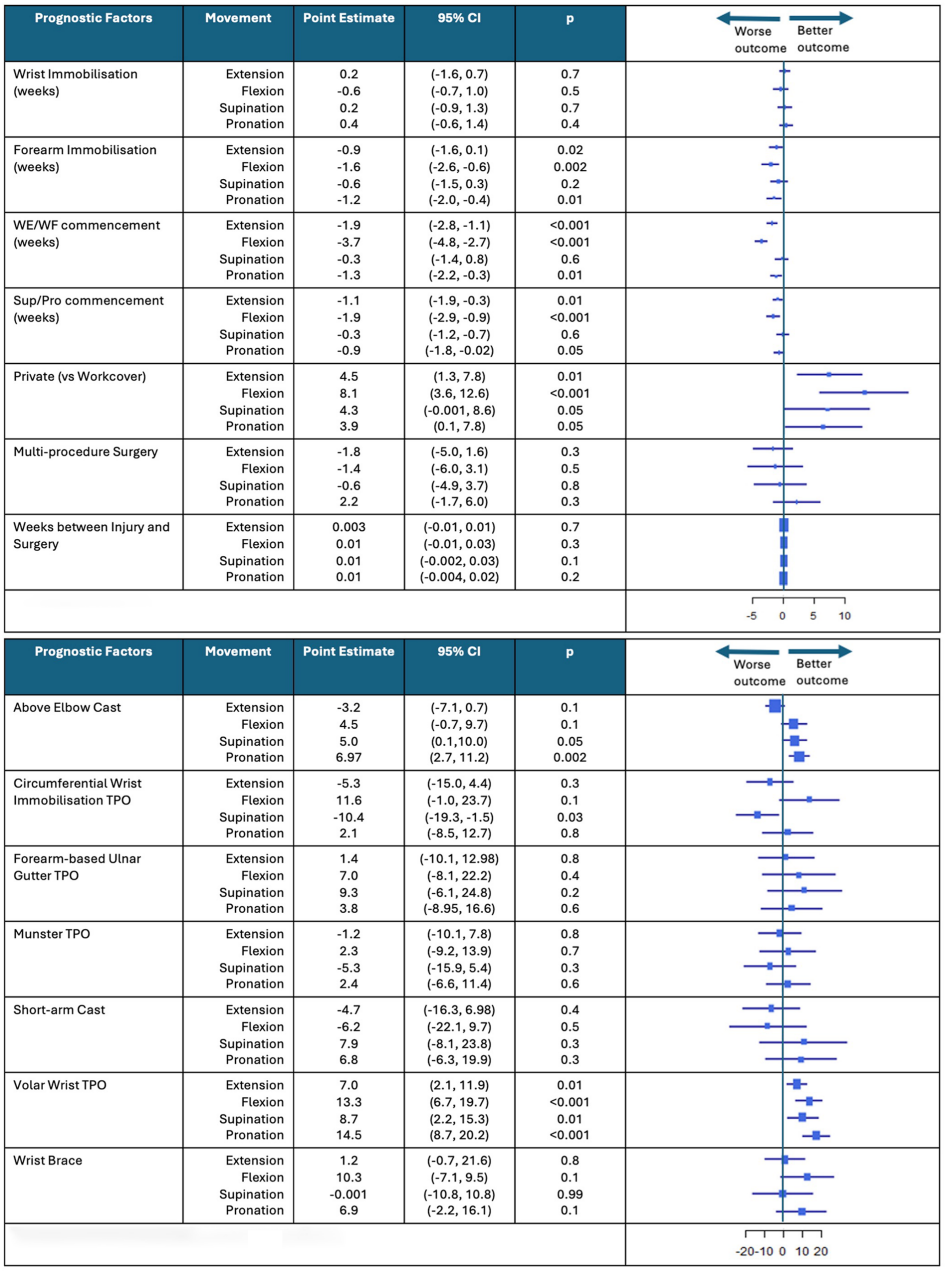
Change in mean range of motion associated with each candidate prognostic factor and immobilization method. *Note.* This figure shows the forest plots that represent the change in mean range of motion for candidate prognostic factors and immobilization method. This figure provides the associated point estimates, 95% CIs, *P*-values, and box plots that show “worse” or “better” outcomes. CI = confidence interval; WE/WF = wrist extension/wrist flexion; TPO = thermoplastic orthosis.

Changes in mean ROM over the duration of recovery associated with each candidate prognostic factor and immobilization method are illustrated in Figure 4. There were several significant candidate prognostic factors that predicted ROM progression in this population. Private patients had on average 4.5° better wrist extension (*P* = .01; 95% CI, 1.3-7.8), 8.1° better wrist flexion (*P* < .001; 95% CI, 3.6-12.6), 4.3° better supination (*P* = .05; 95% CI, −0.001 to 8.6), and 3.9° better pronation (*P* = .05; 95% CI, 0.1-7.8) than Workcover patients throughout recovery. Longer forearm immobilization duration predicted 0.9° poorer wrist extension (*P* = .02; 95% CI, −1.6 to −0.1), 1.6° poorer wrist flexion (*P* = .002; 95% CI, −2.6 to −0.6), and 1.2° poorer pronation (*P* = .01; 95% CI, −2.0 to −0.4), whereas wrist immobilization duration did not predict ROM progression. Delayed WE/WF exercise commencement time also predicted 1.9° poorer wrist extension (*P* < .001; 95% CI, −2.8 to −1.1), 3.7° poorer wrist flexion (*P* < .001; 95% CI, −4.8 to −2.7), and 1.3° poorer pronation (*P* = .01; 95% CI, −2.2 to −0.3). Similarly, delayed SUP/PRO exercise commencement time predicted 1.1° poorer wrist extension (*P* = .01; 95% CI, −1.9 to −0.3), 1.9° poorer wrist flexion (*P* < .001; 95% CI, −2.9 to −0.9), and 0.9° poorer pronation (*P* = .05; 95% CI, −1.8 to −0.02).

Patients who received a volar wrist TPO demonstrated 7° improved wrist extension (*P* = .01; 95% CI, 2.1-11.9), 13.3° improved wrist flexion (*P* < .001; 95% CI, 6.7-19.7), 8.7° improved supination (*P* = .01; 95% CI, 2.2-15.3), and 14.5° improved pronation (*P* = < .001; 95% CI, 8.7-20.2), whereas patients who received an above elbow cast showed 5° improved supination (*P* = .05; 95% CI, 0.1-10.0) and 6.97° improved pronation (*P* = .002; 95% CI, 2.7-11.2). On the contrary, patients who received a circumferential wrist immobilization TPO showed 10.4° poorer supination (*P* = .03; 95% CI, −19.3 to −1.5).

### Candidate Prognostic Factors for Grip Strength Progression

**Figure 5.**
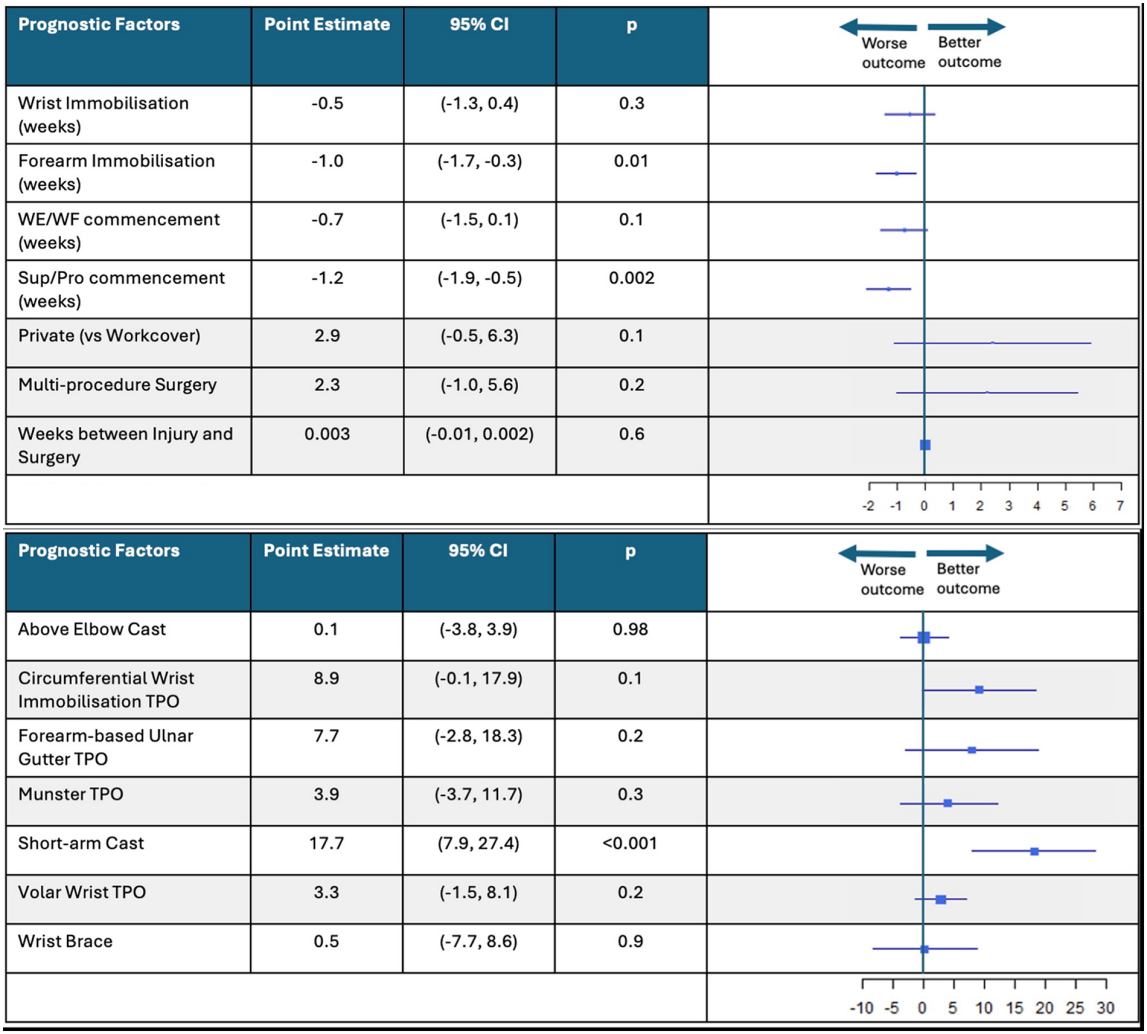
Change in mean grip strength associated with each candidate prognostic factor and immobilization method. *Note.* This figure shows the forest plots that represent the change in mean grip strength for candidate prognostic factors and immobilization method. This figure provides the associated point estimates, 95% CIs, *P*-values, and box plots that show “worse” or “better” outcomes. CI = confidence interval; WE/WF = wrist extension/wrist flexion; TPO = thermoplastic orthosis.

There were 3 candidate prognostic factors that predicted grip strength progression over the duration of recovery in this population (Figure 5). Patients who received longer forearm immobilization duration demonstrated 1 kg poorer grip strength (*P* = .01; 95% CI, −1.7 to −0.3), whereas delayed SUP/PRO exercise commencement time resulted in 1.2 kg poorer grip strength (*P* = .002; 95% CI, −1.9 to −0.5). Alternatively, patients who received a short-arm cast demonstrated, on average, 17.7 kg higher grip strength throughout the recovery period compared with other immobilization methods (*P* < .001; 95% CI, 7.9-27.4). No other immobilization method predicted grip strength.

### Candidate Prognostic Factors for Pain Progression

**Figure 6.**
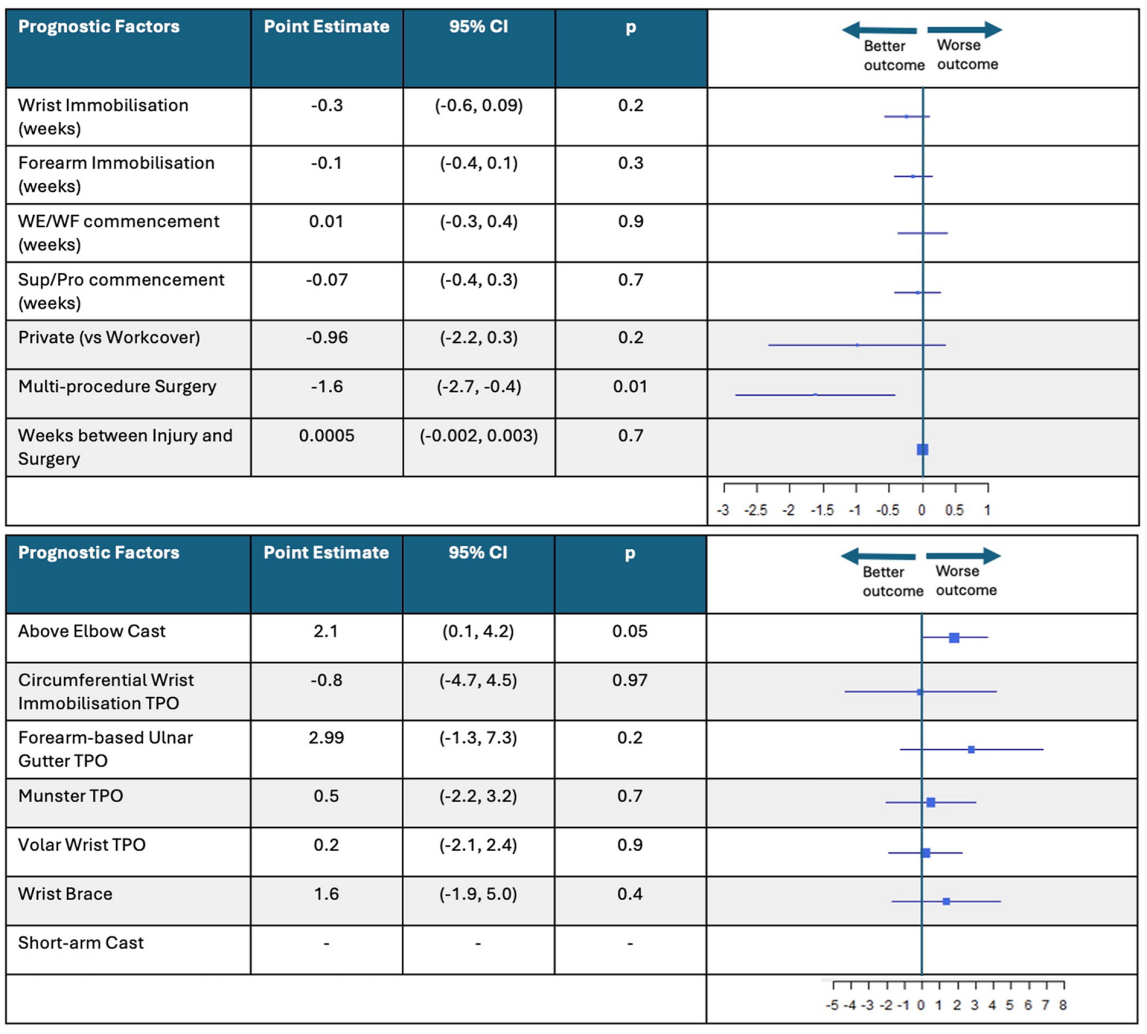
Change in pain scores associated with each candidate prognostic factor and immobilization method. *Note.* This figure shows the forest plots that represent the change in mean pain for candidate prognostic factors and immobilization method. This figure provides the associated point estimates, 95% CIs, *P*-values, and box plots that show “worse” or “better” outcomes. CI = confidence interval; WE/WF = wrist extension/wrist flexion; TPO = thermoplastic orthosis.

One candidate prognostic factor and one immobilization method predicted pain progression over the duration of recovery in this population (Figure 6). Patients in the multiprocedure group reported pain that was 1.6 points lower than patients in the single procedure group (*P* = .01; 95% CI, −2.7 to −0.4), whereas patients who received an above elbow cast reported pain that was 2.1 points higher (*P* = .05; 95% CI, 0.1-4.2).

### Adverse Events

**Figure 7.**
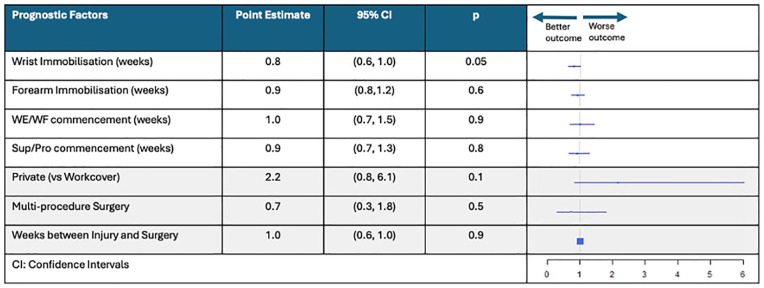
Forest plot to show the risk of triangular fibrocartilage complex rupture associated with each candidate prognostic factor. *Note.* This figure shows the forest plots that represent rupture risk for candidate prognostic factors. This figure provides the associated point estimates, 95% CIs, *P*-values, and box plots that show “worse” or “better” outcomes. CI = confidence interval; TPO = thermoplastic orthosis.

Only one candidate prognostic factor predicted TFCC rupture in this population (Figure 7). Patients who received a shorter wrist immobilization period were more likely to rupture (*P* = .05; 95% CI, 0.6-1.00).

## Discussion

This retrospective case-series has comprehensively described the demographic data, rehabilitation characteristics, trajectory for recovery, and prognostic factors following TFCC foveal repair surgery. Several demographic and rehabilitation characteristics were identified that predicted patient outcomes. Longer forearm immobilization predicted poorer ROM and grip strength progression, which may be explained by increased postoperative stiffness and additional scar tissue associated with longer immobilization periods.^20^ However, longer wrist immobilization did not predict poorer ROM progression. This discrepancy may be explained by the TFCC’s heightened role in stabilizing forearm rotation, compared with WE/WF. In contrast, a *shorter* wrist immobilization period was found to be the only characteristic that predicted an increased risk of TFCC rupture following foveal repair surgery, suggesting that wrist motion is also an important factor to consider after TFCC repair. It seems that there is a delicate balance between benefits and risks, with shorter immobilization periods following surgery leading to greater ROM and grip strength but with an increased risk of rupture. These findings support a staged postoperative rehabilitation approach that commences with controlled forearm ROM exercises in the earlier (acute) stages of recovery, while immobilizing the wrist until the later (subacute) stages. This may be facilitated by the implementation of an immobilization method that restricts wrist motion, while allowing some forearm rotation, such as a volar wrist TPO or short-arm cast, both of which were found in this study to predict improved ROM and grip strength, respectively.

Workcover status was found to predict ROM progression, but not grip strength. Workcover patients demonstrated poorer ROM progression compared with private patients; however, grip strength progression was the same for both groups. Grip strength was consistently measured between clinics using a hand-held dynamometer, which is a standardized outcome measure with strong validity and reliability,^21^ whereas ROM was measured using a standard wrist goniometer, which can be influenced by several therapist and patient factors.^22,23^ This finding suggests that change in grip strength may be a more indicative outcome when assessing progress in Workcover patients following TFCC foveal repair surgery.

The TFCC rupture rate identified in this study was 10.5%, which was higher than a previously reported scoping review of TFCC rehabilitation (1%),^24^ though similar to the rupture rate reported in a survey of Accredited Hand Therapists (11%).^6^ This suggests that an accurate rupture rate following TFCC foveal repair surgery may be approximately 10% to 11%. This is comparable to other ligament repair surgery of the wrist, such as the scapholunate ligament, which has been reported to have a surgical failure rate of 14% to 17%.^25,26^

### Strengths

This study possessed many strengths. Advanced statistical analysis included Linear Mixed Effects and Logistic Regression Models, facilitating comprehensive examination of the data set, incorporating adjustments for the prognostic factors age and sex, as well as accounting for varying time points and individual differences. The multicenter approach that included 7 private practice hand therapy clinics nationwide yielded a relatively large patient sample, which increased the robustness and validity of the findings. A clearly defined inclusion criteria and the dual researcher data extraction process also increased robustness by ensuring that data were accurately entered into the database prior to analyses, minimizing bias and error.

### Limitations

Due to the large number of candidate predictors being examined, there is an increased risk of type 1 error. In addition, although statistical significance was identified, some of the differences may not be clinically important. Thus, the findings of this study need to be replicated in further prospective longitudinal studies and subsequent meta-analysis. The study data were specific to an Australian private practice setting and as such the findings may not be generalizable to a public hospital or international setting. The initial plan in this study was to include patient-rated outcome measures, such as the Disabilities of the Arm, Shoulder and Hand^27^ or Patient-Rated Wrist and Hand Evaluation.^28^ However, following data extraction it was identified that insufficient patient-rated outcome data were available to conduct an accurate and meaningful analysis. In addition, data regarding strengthening approach or proprioceptive retraining were also limited. Inconsistencies were sometimes noted in the detail and completeness of clinical notes. This lack of standardization made it difficult to accurately determine key factors such as the duration of therapy. To address these challenges, we recommend the development of a core outcome set to enhance consistency in data collection and reporting. Furthermore, accurate clinical record keeping is essential not only for medico-legal purposes but also to effectively quantify patient recovery over time and guide evidence-based practice. Finally, we did not have access to the surgeons’ operative notes, so the details describing the surgery technique were limited to what the hand therapist had reported. As such, reporting errors through this third-party reporting method cannot be excluded, nor can other confounding factors such as multiple repair techniques.

### Future Research

Recommendations for future research include additional prospective studies, such as cohort studies or randomized control trial, to confirm the findings of this study, for example TFCC rupture rate, and validate the prognostic factors related to ROM and grip strength progression. This may be best achieved using a multicenter approach due to the low relative incidence rate of TFCC foveal repair surgery. Future research may choose to include both academic and practice settings, as well as international study sites, which would increase the generalizability of the findings. Future prospective research should include patient-rated outcome measures, with the development of a core outcome set for TFCC injuries and/or musculoskeletal wrist conditions of interest to hand therapists and surgeons alike. This set should be established using the best available evidence and an international consensus process, to enhance both research and clinical practice.^29^ The development of rehabilitation guidelines for patients following TFCC foveal repair surgery may also be of benefit.

In summary, there were several prognostic factors which were found to predict patient outcomes in this post TFCC foveal repair surgery population. Longer forearm immobilization duration was found to predict poorer ROM and grip strength, whereas a shorter wrist immobilization period increased the risk of TFCC rupture. Workcover patients demonstrated poorer ROM progression than private patients; however, this did not predict grip strength progression. These insights are hoped to guide clinical decision-making and lead to the development of rehabilitation guidelines for patients following TFCC foveal repair surgery.

## Supplemental Material

sj-docx-1-han-10.1177_15589447251325821 – Supplemental material for Predictive Factors for Patient Recovery Following Triangular Fibrocartilage Foveal Repair Surgery: A Retrospective Case-Series Supplemental material, sj-docx-1-han-10.1177_15589447251325821 for Predictive Factors for Patient Recovery Following Triangular Fibrocartilage Foveal Repair Surgery: A Retrospective Case-Series by Luke McCarron, Brooke K Coombes, Randy Bindra, Brett Dyer, Steven Watson and Leanne Bisset in HAND

sj-docx-2-han-10.1177_15589447251325821 – Supplemental material for Predictive Factors for Patient Recovery Following Triangular Fibrocartilage Foveal Repair Surgery: A Retrospective Case-Series Supplemental material, sj-docx-2-han-10.1177_15589447251325821 for Predictive Factors for Patient Recovery Following Triangular Fibrocartilage Foveal Repair Surgery: A Retrospective Case-Series by Luke McCarron, Brooke K Coombes, Randy Bindra, Brett Dyer, Steven Watson and Leanne Bisset in HAND

sj-docx-3-han-10.1177_15589447251325821 – Supplemental material for Predictive Factors for Patient Recovery Following Triangular Fibrocartilage Foveal Repair Surgery: A Retrospective Case-Series Supplemental material, sj-docx-3-han-10.1177_15589447251325821 for Predictive Factors for Patient Recovery Following Triangular Fibrocartilage Foveal Repair Surgery: A Retrospective Case-Series by Luke McCarron, Brooke K Coombes, Randy Bindra, Brett Dyer, Steven Watson and Leanne Bisset in HAND

sj-jpg-4-han-10.1177_15589447251325821 – Supplemental material for Predictive Factors for Patient Recovery Following Triangular Fibrocartilage Foveal Repair Surgery: A Retrospective Case-Series Supplemental material, sj-jpg-4-han-10.1177_15589447251325821 for Predictive Factors for Patient Recovery Following Triangular Fibrocartilage Foveal Repair Surgery: A Retrospective Case-Series by Luke McCarron, Brooke K Coombes, Randy Bindra, Brett Dyer, Steven Watson and Leanne Bisset in HAND

sj-jpg-5-han-10.1177_15589447251325821 – Supplemental material for Predictive Factors for Patient Recovery Following Triangular Fibrocartilage Foveal Repair Surgery: A Retrospective Case-Series Supplemental material, sj-jpg-5-han-10.1177_15589447251325821 for Predictive Factors for Patient Recovery Following Triangular Fibrocartilage Foveal Repair Surgery: A Retrospective Case-Series by Luke McCarron, Brooke K Coombes, Randy Bindra, Brett Dyer, Steven Watson and Leanne Bisset in HAND
