# Supplementary material for: Predictive Factors for Patient Recovery Following Triangular Fibrocartilage Foveal Repair Surgery: A Retrospective Case-Series
Source: Hand (N Y). 2025 Mar 31;21(4):645–56. doi: 10.1177/15589447251325821 (PMC11959570; doi:10.1177/15589447251325821)
Supplement: sj-docx-1-han-10.1177_15589447251325821 – Supplemental material for Predictive Factors for Patient Recovery Following Triangular Fibrocartilage Foveal Repair Surgery: A Retrospective Case-Series [file sj-docx-1-han-10.1177_15589447251325821.docx]

Supplementary Figure Labels

Supplementary Figure 1: Post-operative Wrist and Forearm Immobilisation Duration. This figure demonstrates the post-operative wrist and forearm immobilization duration period, as documented in the clinical notes, organised according to the week post-surgery.

Supplementary Figure 2: Post-operative Range of Motion Exercise Commencement Week. This figure demonstrates the post-operative range of motion exercise commencement week, as documented in the clinical notes, organised according to the week post-surgery. Separate values have been provided for each respective joint and movement plane, wrist extension/flexion, wrist ulnar/radial deviation, forearm supination/pronation, and elbow extension/flexion.
