# Supplementary material for: Predictive Factors for Patient Recovery Following Triangular Fibrocartilage Foveal Repair Surgery: A Retrospective Case-Series
Source: Hand (N Y). 2025 Mar 31;21(4):645–56. doi: 10.1177/15589447251325821 (PMC11959570; doi:10.1177/15589447251325821)
Supplement: sj-docx-2-han-10.1177_15589447251325821 – Supplemental material for Predictive Factors for Patient Recovery Following Triangular Fibrocartilage Foveal Repair Surgery: A Retrospective Case-Series [file sj-docx-2-han-10.1177_15589447251325821.docx]

Supplementary Table 1. Australian Standard Classifications of Occupations (ASCO)

| Occupation Category | Total Group  (n= 210) |
| --- | --- |
| 1. Managers and Administrators  2. Professionals  3. Associate Professions  4. Tradespersons and Related Workers  5. Advanced Clerical and Service Workers  6. Clerical, Sales and Service Workers  7. Production and Transport Workers  8. Elementary Clerical, Sales and Service Workers  9. Labourers and Related Workers  10. Nil – Retired or Not Employed  11. Homecare or Housewife  *Student | 11 (5%)  37 (17.5%)  35 (16.5%)  34 (16%)  15 (7%)  3 (1.5%)  4 (2%)  22 (10.5%)  10 (5%)  20 (9.5%)  2 (1%)  17 (8%) |
| *A student group was added to represent high school or university/college students who met the inclusion criteria. | |
