# Supplementary material for: Predictive Factors for Patient Recovery Following Triangular Fibrocartilage Foveal Repair Surgery: A Retrospective Case-Series
Source: Hand (N Y). 2025 Mar 31;21(4):645–56. doi: 10.1177/15589447251325821 (PMC11959570; doi:10.1177/15589447251325821)
Supplement: sj-docx-3-han-10.1177_15589447251325821 – Supplemental material for Predictive Factors for Patient Recovery Following Triangular Fibrocartilage Foveal Repair Surgery: A Retrospective Case-Series [file sj-docx-3-han-10.1177_15589447251325821.docx]

Supplementary Table 2: Surgical Procedure List

| Surgical Procedures | Frequency  (n=210) | Percentage |
| --- | --- | --- |
| TFCC Repair | 125 | 59.5 |
| TFCC Repair + USO | 21 | 10.0 |
| TFCC Repair + CTR | 10 | 4.8 |
| TFCC Repair + E/O Ganglion | 7 | 3.3 |
| TFCC Repair + E/O Ulnar Styloid | 7 | 3.3 |
| TFCC Repair + UN Transposition | 4 | 1.9 |
| TFCC Repair + ECU Stabilisation | 4 | 1.9 |
| TFCC Repair + UN Release | 3 | 1.4 |
| TFCC Repair + USO + CTR | 3 | 1.4 |
| TFCC Repair + SLL Repair | 2 | 1.0 |
| TFCC Repair + E/O Pisiform | 2 | 1.0 |
| TFCC Repair + De Quervain’s Release | 2 | 1.0 |
| TFCC Repair + Trigger Finger Release | 2 | 1.0 |
| TFCC Repair + CTR + ECU Stabilisation | 1 | 0.5 |
| TFCC Repair + CTR + R/O Plate | 1 | 0.5 |
| TFCC Repair + CTR + SLL Debridement | 1 | 0.5 |
| TFCC Repair + CTR + E/O Scaphoid AVN + E/O Ulnar Styloid | 1 | 0.5 |
| TFCC Repair + De Quervain’s Release + R/O Plate | 1 | 0.5 |
| TFCC Repair + E/O Ganglion + E/O Pisiform + CTR | 1 | 0.5 |
| TFCC Repair + E/O Pisiform + CTR | 1 | 0.5 |
| TFCC Repair + E/O Ulnar Styloid + R/O Plate | 1 | 0.5 |
| TFCC Repair + ECU Stabilisation + SLL Capsulodesis | 1 | 0.5 |
| TFCC Repair + LTL Debridement | 1 | 0.5 |
| TFCC Repair + LTL Repair | 1 | 0.5 |
| TFCC Repair + Scaphoid ORIF + USO | 1 | 0.5 |
| TFCC Repair + SLL Repair + UN Release | 1 | 0.5 |
| TFCC Repair + Thumb UCL Repair | 1 | 0.5 |
| TFCC Repair + UN Transposition + UN Release (Guyon's Canal) | 1 | 0.5 |
| TFCC Repair + USO + Scaphoid ORIF | 1 | 0.5 |
| TFCC Repair + Wrist Fusion + E/O Pisiform | 1 | 0.5 |
| TFCC Repair + R/O Plate + CTR + Trigger Thumb Release + Trigger Finger Release + E/O Ulnar Styloid | 1 | 0.5 |
| Total | 210 | 100.0 |
| List of Abbreviations - USO: ulnar shortening osteotomy. CTR: carpal tunnel release. E/O: excision of. UN: ulnar nerve. SLL: scapholunate ligament. ECU: extensor carpi ulnaris. R/O: removal of. AVN: avascular necrosis. LTL: lunotriquetral ligament. ORIF: open reduction internal fixation. UCL: ulnar collateral ligament. | | |
