# Supplementary figures and images for: Predictive Factors for Patient Recovery Following Triangular Fibrocartilage Foveal Repair Surgery: A Retrospective Case-Series
Source: Hand (N Y). 2025 Mar 31;21(4):645–56. doi: 10.1177/15589447251325821 (PMC11959570; doi:10.1177/15589447251325821)

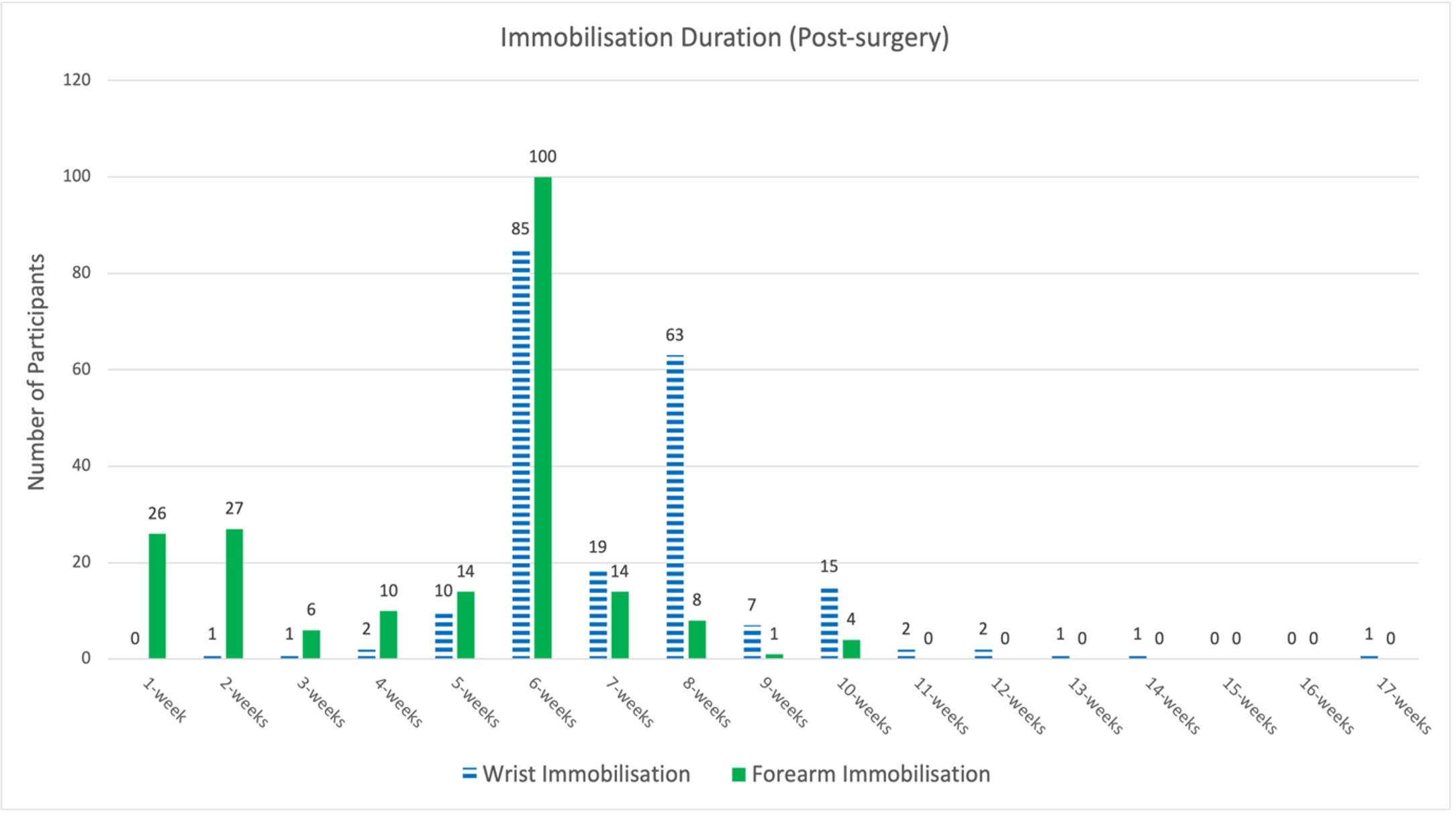

Supplement: sj-jpg-4-han-10.1177_15589447251325821 – Supplemental material for Predictive Factors for Patient Recovery Following Triangular Fibrocartilage Foveal Repair Surgery: A Retrospective Case-Series [file sj-jpg-4-han-10.1177_15589447251325821.jpg]

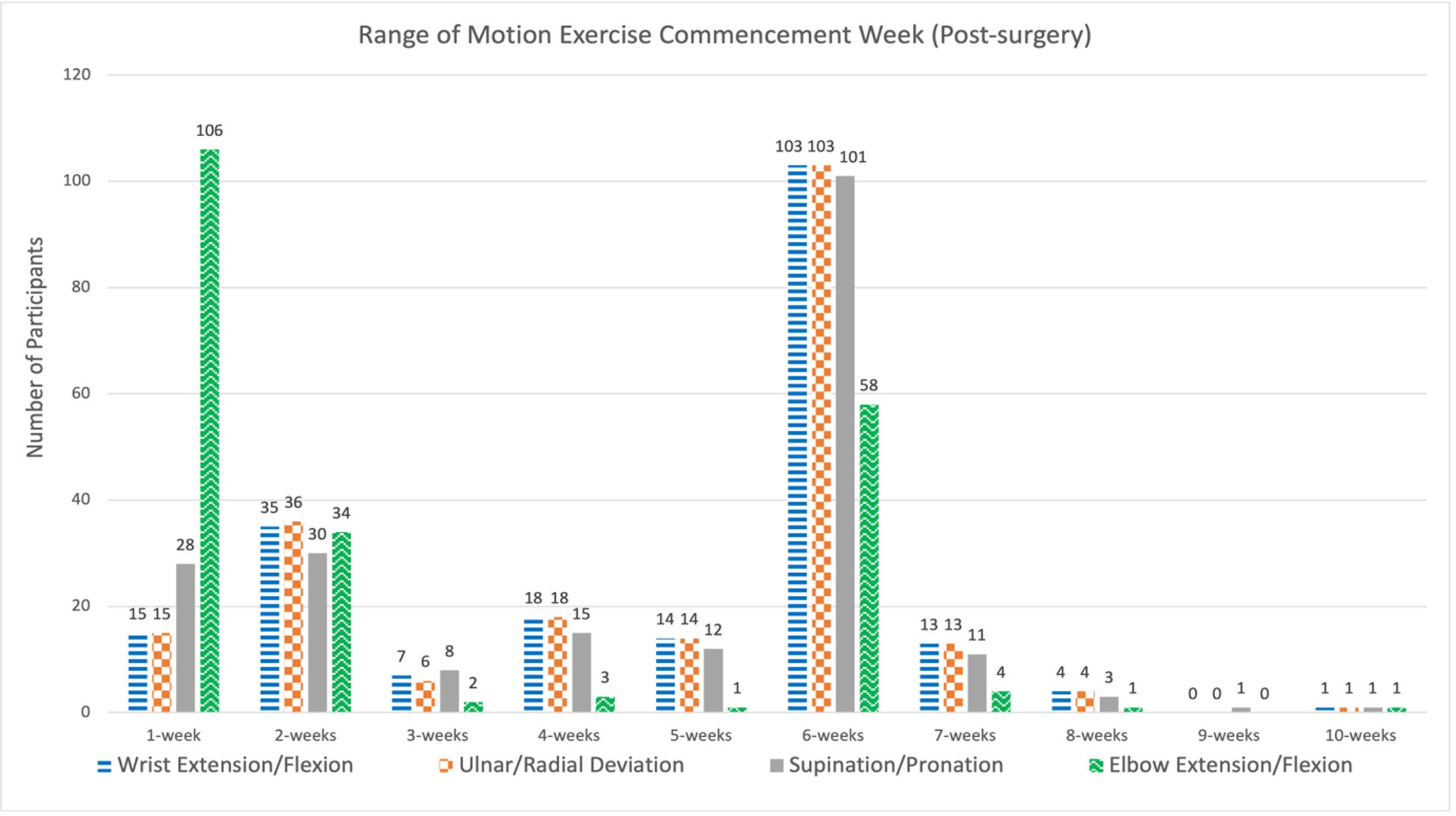

Supplement: sj-jpg-5-han-10.1177_15589447251325821 – Supplemental material for Predictive Factors for Patient Recovery Following Triangular Fibrocartilage Foveal Repair Surgery: A Retrospective Case-Series [file sj-jpg-5-han-10.1177_15589447251325821.jpg]
